# Can Seismocardiogram Fiducial Points Be Used for the Routine Estimation of Cardiac Time Intervals in Cardiac Patients?

**DOI:** 10.3389/fphys.2022.825918

**Published:** 2022-03-18

**Authors:** Zeynep Melike Işilay Zeybek, Vittorio Racca, Antonio Pezzano, Monica Tavanelli, Marco Di Rienzo

**Affiliations:** ^1^WeST Lab, IRCCS Fondazione Don Carlo Gnocchi ONLUS, Milan, Italy; ^2^Cardiac Rehabilitation Unit, IRCCS Fondazione Don Carlo Gnocchi ONLUS, Milan, Italy

**Keywords:** telerehabilitation, telemedicine, cardiac mechanics, heart failure, myocardial infarction, heart transplant

## Abstract

The indexes of cardiac mechanics can be derived from the cardiac time intervals, CTIs, i.e., the timings among the opening and closure of the aortic and mitral valves and the Q wave in the ECG. Traditionally, CTIs are estimated by ultrasound (US) techniques, but they may also be more easily assessed by the identification of specific fiducial points (FPs) inside the waveform of the seismocardiogram (SCG), i.e., the measure of the thorax micro-accelerations produced by the heart motion. While the correspondence of the FPs with the valve movements has been verified in healthy subjects, less information is available on whether this methodology may be routinely employed in the clinical practice for the monitoring of cardiac patients, in which an SCG waveform distortion is expected because of the heart dysfunction. In this study we checked the SCG shape in 90 patients with myocardial infarction (MI), heart failure (HF), or transplanted heart (TX), referred to our hospital for rehabilitation after an acute event or after surgery. The SCG shapes were classified as traditional (T) or non-traditional (NT) on whether the FPs were visible or not on the basis of nomenclature previously proposed in literature. The T shape was present in 62% of the patients, with a higher ∓ prevalence in MI (79%). No relationship was found between T prevalence and ejection fraction (EF). In 20 patients with T shape, we checked the FPs correspondence with the real valve movements by concomitant SCG and US measures. When compared with reference values in healthy subjects available in the literature, we observed that the Echo vs. FP differences are significantly more dispersed in the patients than in the healthy population with higher differences for the estimation of the mitral valve closure (−17 vs. 4 ms on average). Our results indicate that not every cardiac patient has an SCG waveform suitable for the CTI estimation, thus before starting an SCG-based CTI monitoring a preliminary check by a simultaneous SCG-US measure is advisable to verify the applicability of the methodology.

## Introduction

The assessment of cardiac mechanics is essential for the evaluation of the heart performance in health and disease (Lilly and Braunwald, 2012). Some indexes of the heart mechanical function can be derived from the measure of the cardiac time intervals (CTIs) (Oh and Tajik, 2003). They provide information on systolic (Weissler et al., 1968) and diastolic function of the heart (Mancini et al., 1982) on the basis of timings among opening and closure of the aortic and mitral valves, AO, AC, MO, MC, respectively, and the Q wave in the ECG. The most common CTIs are the pre-ejection period (PEP) (defined as the Q-AO time delay), the isovolumic contraction time (IVCT) (MC-AO time delay), the left ventricular ejection time (LVET) (AO-AC time delay), and the isovolumic relaxation time (IVRT) (AC-MO time delay).

Traditionally, CTIs are estimated by ultrasound (US) techniques (Myreng and Smiseth, 1990; Reant et al., 2010; Biering-Sørensen et al., 2013). By this approach, the measures are sporadic and should be taken by an expert operator with the subject in a controlled condition and often in a clinical setting.

**FIGURE 1:**
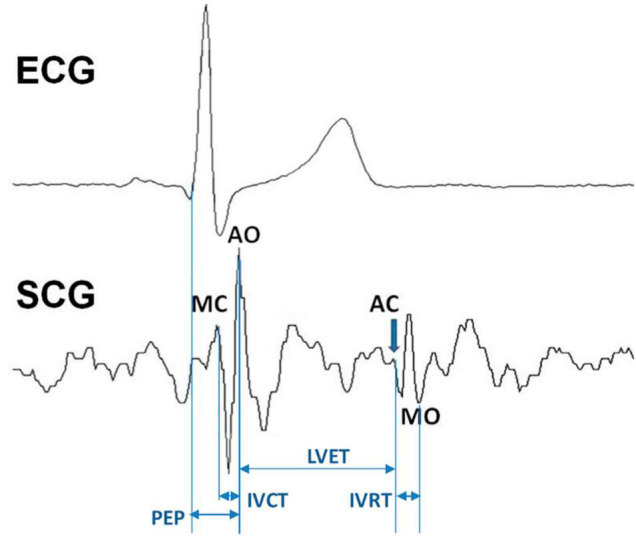
The seismocardiogram (SCG) signal, the localization of the fiducial points associated with the opening and closure of the aortic and mitral valve, its relation with the ECG waveform, and the schematization of how cardiac time intervals are derived. See text for abbreviations.

However, CTIs may be also evaluated by the seismocardiogram (SCG) (Marcus et al., 2007; Neary et al., 2011; Di Rienzo et al., 2013; Inan et al., 2015; Shafiq et al., 2016; Khosrow-Khavar et al., 2017). This signal is the measure of the minute thorax acceleration produced by the heart motion and can be assessed by comfortable and low-priced wearable devices in daily life (Di Rienzo et al., 2013; Inan et al., 2015). Although SCG is a three-dimensional signal, only the dorso-ventral component of acceleration is usually considered for its assessment and in our analysis, we comply with this traditional approach. As shown in Figure 1, the typical SCG waveform in healthy subjects is characterized by a sequence of peaks and valleys. On the basis of simultaneous US and SCG recordings, it has been observed that some of these patterns can be taken as fiducial points (FPs) associated with the AO, AC, MO, and MC valve events, from which CTIs may be derived (Salerno and Zanetti, 1990; Crow et al., 1994; Sørensen et al., 2018; Dehkordi et al., 2019).

Due to the advantages of this approach (easy use and the possibility of prolonged monitoring in daily life), its translation to the clinical practice would be highly desirable. This would open the possibility to monitor the mechanical performance of cardiac patients remotely and for a long period as a part of telemedicine and telerehabilitation programs.

In this perspective, it should be considered that in cardiac patients, changes in the SCG waveform can be expected because of heart dysfunction. Therefore for a clinical application of the methodology, it should be preliminarily verified if the distortion of the SCG waveform caused by the disease might prevent the identification of the FPs from which CTIs are derived. Indeed, any change in the heart muscle structure or function, dyssynchrony among contractions and relaxations of the left and right heart, rhythm disturbances, or valve defects, is likely to result in alterations in the heart movements and thus in distorted SCG waveforms. Moreover, patients may undergo cardiac surgery [e.g., for a coronary bypass grafting, or heart transplant (TX)] and this implies the opening and closure of the thorax with a likely modification of the inner tissues and creation of scar tissue, thus leading to possible changes in the transmission of vibrations from the heart to the external surface of the chest, where SCG is detected.

Actually, since the first paper proposing the SCG (at that time termed precordial ballistocardiogram) (Mounsey, 1957), it has been observed that altered SCG waves may occur in patients suffering from different heart diseases, with many subsequent confirmations (Salerno and Zanetti, 1990; Wilson et al., 1993; Crow et al., 1994; Korzeniowska-Kubacka et al., 2006; Becker et al., 2014; Inan et al., 2018; Schmidt et al., 2021).

However, in literature, there are research studies in which CTIs are derived from the patients’ SCG data, such as subjects with hypertrophic or dilated cardiomyopathy, aortic valve disease, ischemic heart disease, and heart failure (HF) (Crow et al., 1994; Korzeniowska-Kubacka et al., 2006, 2007; Marcus et al., 2007; Becker et al., 2014; Khosrow-Khavar et al., 2017). Thus, it appears that distortions in the SCG shape caused by the heart diseases exist but do not invariably prevent the FPs identification.

It remains to be clarified on which proportion of cardiac patients this approach may be employed in the clinical practice, out of the research laboratory. This pilot study aimed to focus on this issue.

In particular, we evaluated the applicability of the SCG-based CTI monitoring in a sample of patients referred to our cardiac rehabilitation hospital. We considered patients suffering from myocardial infarction (MI) or HF, and those after TX. In these patients we investigated (a) if their SCG shapes allow the identification of traditional FPs (from which CTIs are derived); and, if so, (b) whether the FPs are still correlated with valve events, as in healthy subjects, so that CTIs can be correctly estimated.

## Materials and Methods

### Subjects

In this study, we analyzed data of 90 cardiac patients (67 men and 23 women; age mean ± *SD* and range: 64.8 ± 14.0 years, 25–87 years) collected in the frame of two previous investigations. The dataset included 34 patients with MI, 49 with HF and 7 after TX referred to our hospital in the period between 2016 and 2019 for cardiac rehabilitation after an acute event or surgical intervention. We refrained from imposing exclusion criteria on the basis of disease stage, treatments, and co-morbidities to reflect the typical patient complexity of a real clinical scenario. The breakdown of the recruited patients based on the most frequent co-morbidities is shown in Table 1. The experimental protocols were approved by our Ethical committee (no. 7_14/12/2016, 5_15/02/2019) and patients signed written informed consent before participating in the investigations.

**TABLE 1:** Breakdown of patients based on the most frequent co-morbidities.

Observed co-morbidities	No. patients
Anemia	23
Chronic kidney disease	15
Chronic obstructive pulmonary disease	18
Diabetes	20
Hypertension	31
Obesity	5
Neurological degenerative disease	4
Peripheral neuropathy	2
Peripheral obliterative arteriopathy	7
Previous neoplasms	12
Previous stroke	5
Thyroid disease	16

### Data Collection and Analysis

**FIGURE 2:**
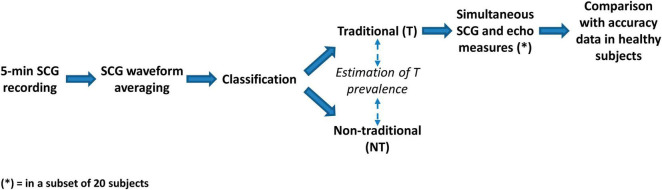
Scheme of the study.

The overall data flow of the study consists of the following steps (Figure 2):

- In each subject a 5-min ECG-SCG recording was made.
  - The individual average SCG waveform was derived.
  - Each average waveform was classified as traditional (T) or non-traditional (NT) by visual inspection.
  - From this classification, the prevalence of T was derived over the whole population, separately for MI, HF, and TX patients, and as a function of the ejection fraction (EF).
  - In a subset of patients inside the T class (*n* = 20), the correspondence of the FPs with the real opening and closure of the aortic and mitral valve was checked by simultaneous SCG and US measures.
  - The obtained results were then compared with the accuracy values observed in healthy subjects and reported in the literature. To the best of our knowledge, the wider study in this area was by Sørensen et al. (2018), and thus we considered their data as a reference for our comparison.

A detailed description of each step is reported hereafter.

In each patient, ECG and SCG were simultaneously recorded for a period of 5 min in lying position using the MagIC-SCG (MS) custom device. Details on this system have been previously published (Di Rienzo et al., 2018a). In short, MS consisted of a miniaturized electronic module for the collection and storage of one ECG lead, 3d accelerations, 3d rotations, and respiratory rate. The system was originally designed for the use in space, and had been exploited for experiments aboard the International Space Station during crew sleep (Di Rienzo et al., 2018b). In the space application, the system was connected to a sensorized vest embedding textile electrodes, while in this study it was connected to traditional gelled ECG adhesive electrodes. SCG was collected by an external wired probe containing an additional accelerometer and connected to MS. As mentioned in the section “Introduction,” we considered the dorso-ventral component of the signal, corresponding to the *z*-axis of our accelerometer. ECG and SCG data were digitized on 14-bit @200 Hz and locally stored on a memory card.

The SCG tracings were filtered by zero-phase band-pass Butterworth filter (third order, 5–40 Hz bandwidth), and for each patient, a data window, including at least six good quality beats, was selected. The SCG waves inside the window were ensemble-averaged, using the ECG R peak for synchronization, thus obtaining the patient’s typical SCG waveform. In four patients suffering from frequent ectopic beats, only a single sinus beat was selected to avoid smearing of the SCG shape due to the inclusion of irregular beats.

Individual anonymized SCG waveforms were visually checked by two experts which independently provided their classification in the T or NT class. In the case of discordant evaluations, a final agreement was obtained by an additional joint check. Classification was based on the presence or absence, respectively, of the typical patterns characterizing *all* four FPs associated with MC, AO, AC, and MO, as defined by Crow et al. (1994).

A large body of literature reports that even in healthy subjects, a significant inter- and intra-subject variability in the SCG shape may occur because of different factors, such as posture and respiration (Crow et al., 1994; Di Rienzo et al., 2013; Inan et al., 2015; Paukkunen et al., 2016; Shafiq et al., 2016; Hansen, 2019; Liu et al., 2019; Taebi et al., 2019). Such variabilities make it difficult to discriminate T from NT waveforms by univocal criteria. Indeed, to the best of our knowledge, no guideline or consensus document exists on this crucial aspect of the SCG analysis. For this reason, notwithstanding its intrinsic limitation, the visual classification of the SCG waveforms is the current standard for the evaluation of the SCG shape (and the current benchmark for the validation of any algorithm processing the SCG signal), thus, we conformed with this practice. It may be expected that our classification will be widely shared for the waveforms close to the reference definition or totally divergent from this, while for the waveforms falling in a “gray zone” in-between the two classes there could be less consensus. To mitigate this unavoidable subjectivity, all SCG shapes considered in the study are shown in the section “Result,” thus leaving the possibility of a personal “readjustment” of our classification in the case of different individual criteria.

In 20 patients within the T class, we also checked if the FPs derived from their SCG waveforms reflected the real valve movements, as in healthy subjects. For this purpose, in each subject, M-mode US images of the aortic and mitral valve were taken by the EPIC 7C US machine (Koninklijke Philips N.V., Amsterdam, Netherlands) using a four-chamber parasternal longitudinal projection. During the US assessment, ECG and SCG were simultaneously recorded by the MS system. For each valve event, we acquired at least 2 images, each displaying the data of 3.5-s, thus usually including from 3 to 5 beats. The sonogram included the plot of the ECG signal simultaneously collected by the US machine. Synchronization between US images and MS data was obtained by a two-step procedure. In the first step, a rough synch was obtained by pushing an event button on the MS system when the echo operator freezed the US image. The event button was controlled by a technician who supervised the MS recording. In the second step, the synchronization was refined by measuring the RR interval of the beats displayed in the sonogram, and searching for the corresponding sequence of intervals in the ECG collected by the MS device.

Three or more good-quality pictures of each valve event were obtained for most patients, with at least one valid picture per subject. In each selected sonogram, an expert cardiologist, blinded to SCG data, measured the timing from the R peak and the target valve event using the MicroDicom viewer.^1^ The resolution of the US images was 2.64 ms per pixel. Multiple measures of the same valve event were averaged for each patient.

For the comparison with the sonograms, the segments of ECG and SCG signals synchronous with the US images were resampled at 1 kHz after interpolation and further analyzed by the same two experts engaged for the T-NT classification. They were blinded to the echo data. For the analysis, they used a custom program that visualizes both signals, and through the use of cursors allows the measurement of the time between the R peak in the ECG signal and the SCG FP associated with each of the considered events (MC, AO, AC, or MO). Cursors were manually positioned by the experts. The difference in the timings taken from the US images and the FPs were then computed to quantify the accuracy of the SCG FP approach in evaluating the real occurrence time of mitral and aortic valve movements.

### Statistics

The prevalence of T waveform was estimated in the whole pool of subjects and separately in the subgroups of patients according to diagnosis (MI, HF, and TX) and EF value (>50%, between 50 and 40%, and <40%). EF was derived from the patient’s clinical records. In five patients, this value was not available.

Given the binomial nature of classification (T vs. NT), the statistical significance of the T prevalence among subgroups was checked by the *G*-test with *p* < 5%.

The significance of the Echo vs. SCG FP differences between patients and healthy subjects was checked by the Welch-test for the mean value and the *F*-test for the SD in both instances with *p* < 5%.

## Results

### Traditional Prevalence

**FIGURE 3:**
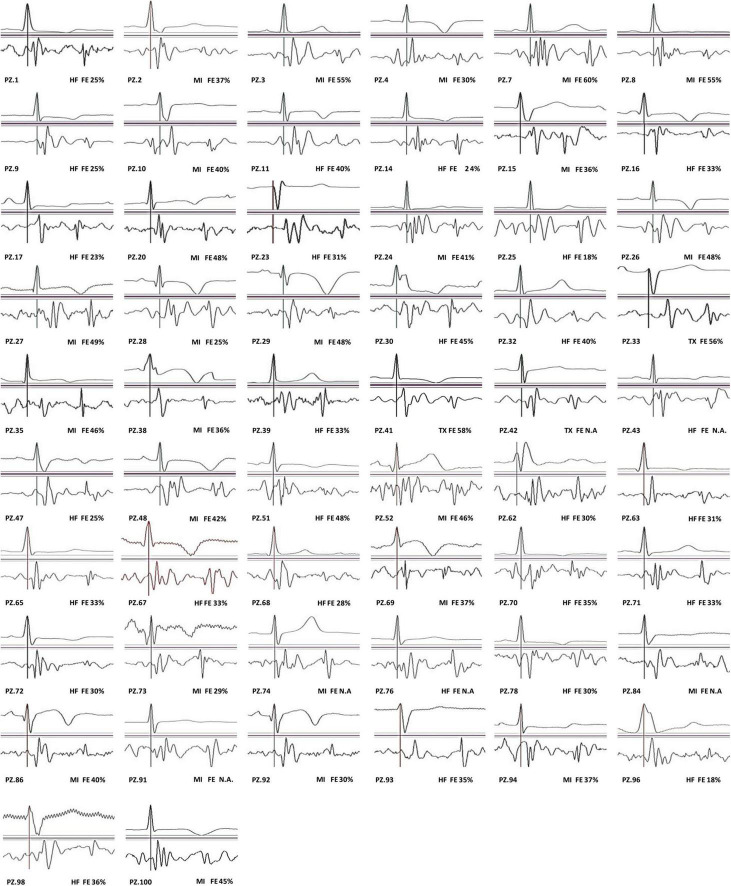
SCG traditional (T) waveforms.

**FIGURE 4:**
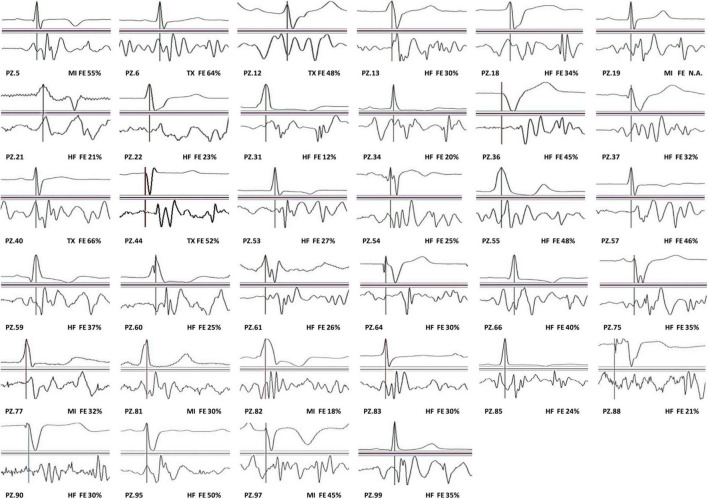
SCG non-traditional (NT) waveforms.

In our population, we observed that 56 out of 90 patients (62%) have traditional SCG shapes. All individual average waveforms classified as T and NT are shown in Figures 3, 4, respectively.

The prevalence of T stratified by diagnosis and EF values are reported in Tables 2, 3.

**TABLE 2:** The T prevalence according to the patient’s diagnosis.

Diagnosis	# Patients	# T waveform	T prevalence
MI	34	26	76%*
HF	49	27	55%
TX	7	3	43%

MI, Myocardial infarction; HF, Heart failure; TX, Heart transplant. * Significant difference vs. HF with p < 0.05.

**TABLE 3:** The T prevalence according to ejection fraction (EF) values.

EF	# Patient	# T waveform	T prevalence
>50% (preserved)	13	8	62%
40–50% (mildly reduced)	21	14	67%
<40% (reduced)	52	31	60%

It appears that T prevalence is markedly higher in MI than in HF and TX patients. No relationship is shown between T prevalence and EF values.

### Fiducial Point Accuracy in the Traditional Class

**FIGURE 5:**
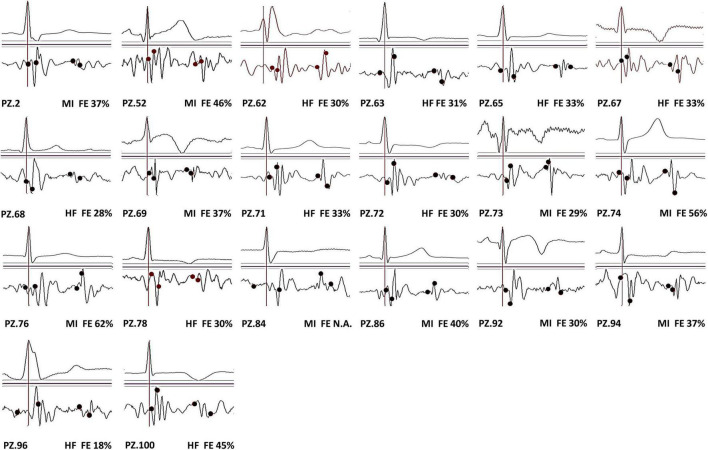
SCG T waveforms from 20 patients with the superimposition of the real location of MC, AO, AC, and MO events (in each panel represented in sequence by the black circles from left to right).

In 20 patients with the T waveform, we investigated the accuracy of the assessment of the opening and closure of the aortic and mitral valve through the SCG FPs by the simultaneous recording of SCG and M-mode US images. Figure 5 illustrates the individual SCG waveforms with the position of the real valve movements detected by echo.

Echo vs. FP differences observed in our patients are detailed in Table 4.

**TABLE 4:** Echo vs. FP differences for each valve event in patients: individual data, group mean, and *SD*.

Echo vs. FP difference [ms]				
Subj #	MC	AO	AC	MO
2	−19	−9	−6	−7
52	−2	−5	−12	−18
62	38	23	−5	−14
63	4	7	−12	−5
65	−34	16	−20	35
67	−5	−7	−3	−10
68	−17	−18	3	22
69	−21	−9	−19	−27
71	3	−2	−5	−1
72	−20	−3	−13	56
73	5	−5	6	−27
74	−29	−12	−10	5
76	−36	−2	−19	−40
78	−6	−16	−9	−46
84	−74	56	26	44
86	−8	−25	−11	−22
92	−17	−22	−42	4
94	−28	−11	−27	−34
96	−77	9	−61	−36
100	2	9	−25	13
MEAN	−17.1	−1.3	−13.2	−5.4
SD	26.2	18.3	17.8	28.3

**FIGURE 6:**
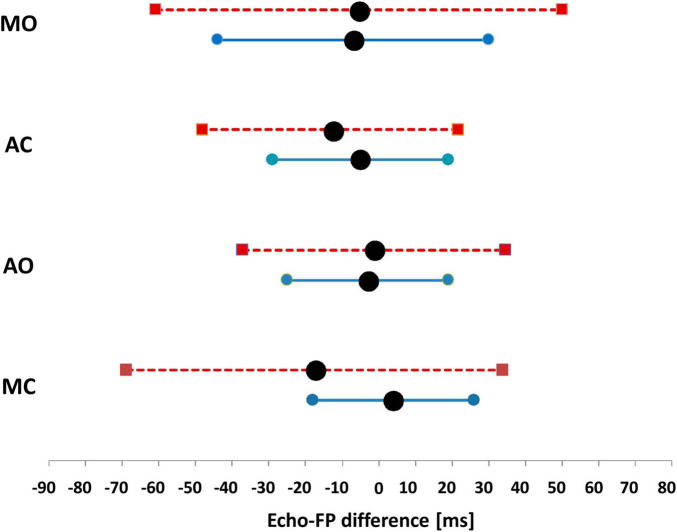
95% reference range of the differences observed in healthy subjects (continuous line), vs. the 95% range observed in the T population of patients (dashed line). Data are separately shown for each FP. Black circles indicate the mean value of each range.

Table 5 reports the differences averaged over the patient group for each valve event, and the corresponding values observed in healthy subjects. In patients, the average difference significantly diverges from the reference for the MC event. On the contrary, the SD of the differences is greater in patients for all the FPs, indicating that the error dispersion is wider in patients. This aspect is better illustrated in Figure 6, where the range of the 95% of the differences (estimated as mean ± 1.96 × SD) observed in healthy subjects is compared with the 95% range observed in patients.

**TABLE 5:** Mean and SD of Echo-FP differences in our 20 patients (_*p*_ subscript) and in the reference healthy subject dataset (_*h*_ subscript).

Fiducial points	Mean [ms]	SD [ms]	Subjects considered
MC_*h*_	4	11	41
MC_*p*_	−17.1^§^	26.2*	20
AO_*h*_	−3	11	39
AO_*p*_	−1.3 (NS)	18.3*	20
AC_*h*_	−5	12	39
AC_*p*_	−13.2 (NS)	17.8*	20
MO_*h*_	−7	19	39
MO_*p*_	−5.4 (NS)	28.3*	20

^§^Mean significantly different from the healthy group with p < 0.05. * SD significantly different from the healthy group with p < 0.05.

Table 6 shows the number and percentage of patients with Echo vs. FP differences falling within the 95% reference range of healthy subjects. It appears that in 76–81% of the T patients AO, AC, and MO events are estimated with the same accuracy than in healthy subjects. Conversely, the estimation of MC is more critical, with 50% of the T patients displaying differences exceeding the 95% range of the reference group. For this valve event, the differences reached values up to −74 and −77 ms (subjects 84 and 96).

**TABLE 6:** 95% range of the Echo vs. FP differences reported in healthy subjects (RRef) and the number of patients with differences falling inside the RRef range.

	MC	AO	AC	MO
95% RRef [ms]	4 ± 22	−3 ± 22	−5 ± 23	−7 ± 37
No. of patients within RRef	10 (50%)	18 (81%)	17 (81%)	17 (76%)

## Discussion

To the best of our knowledge, this is the first study addressing the applicability of the SCG-based estimation of CTIs in the clinical routine.

This was done by checking whether the SCG shape distortion induced by the heart dysfunction might interfere with the identification of the FPs, from which CTIs are derived. For this reason, we classified the SCG waveforms in two classes, T and NT, by visual inspection. To mirror a realistic clinical scenario, in the patient recruitment, we only checked whether the subjects belonged to one of the three target groups (MI, HF, and TX) without imposing any additional exclusion criteria on co-morbidity, gravity of disease, and treatments.

At present, no information is available on the SCG in TX patients, thus our SCG measure in this type of subject, although carried out on a limited sample, represents an additional element of novelty of the study.

### Traditional Shape Prevalence

Our results indicate that in the considered population, little more than half of the recruited patients are characterized by a T shape. This means that for about 40% of the patients, the identification of the FPs can hardly be obtained. When subjects are stratified per diagnosis, the T prevalence is markedly higher for MI than for HF and TX patients, while no relationship was found with the EF values.

For TX patients, our results show that the T prevalence is lower than in MI and HF patients. This is an unexpected finding because our patients did not suffer from any major post-surgery complication, thus, it is assumed that the transplanted heart is correctly functioning and producing T waveforms. The good performance of the new hearts is confirmed in Table 7 where it appears that the average EF value of the TX patients is normal. To explain this apparent contradiction, it can be hypothesized that in TX patients, the heart vibrations are distorted during the transmission through the thorax tissues modified by the surgical opening of the chest (as shown in the “Introduction”). In addition, before TX, only differences in donor vs. recipient body weights are usually checked (Reed et al., 2014), thus some mismatch between the size of the donor heart and the cardiac notch of the recipient patient (i.e., the space between the left and right lungs where the new heart should be accommodated) may occur, with a possible further influence on heart motion and vibration.

**TABLE 7:** Average EF values for each subgroup of patients.

Diagnosis	Average EF
MI	43%
HF	30%
TX	57%

### Echo-Fiducial Points Differences

In 20 patients with the T waveform, the Echo vs. FP differences in the estimation of the valve movements were computed. Over the group, the greater mean differences were observed in the estimation of MC and AC, and their negative values indicate that in our patients these valve movements tend to occur on average before the corresponding FP. The lowest average bias was observed for the AO event (−1 ms). Considering the *SD* values of the differences, it appears that the error dispersion over the group is higher for MC and MO than for AO and AC. This means that there is a larger inter-subject variability in the accuracy of timings associated with the mitral valve than in those related to the aortic valve.

### Comparison With Healthy Subjects

In our study, we compared the error of the Echo vs. FP measures in patients and healthy subjects. Significant discrepancies were observed in the average error for MC (−17.1 ms in patients vs. 4 ms in healthy subjects), and in the error dispersion for all four FPs (always wider in patients). For the MC event, a noticeable fraction of the T patients (50%) displays errors exceeding the 95% range of the reference group. For AO and AC events, the number of patients exceeding the benchmark was 19%, and for MO 24%.

Our data indicate that even if the patient’s SCG waveform is close to the traditional shape, the accuracy in the estimation of the valve movements by FPs may be lower than that observed in healthy subjects. This aspect is particularly evident in the MC estimation, but is somewhat present also in the estimation of the remaining valve events.

### Individual Fiducial Point Identification

For our waveform classification, we considered the simultaneous presence of four FPs. This was done because they are necessary to estimate the CTIs exploring systolic and diastolic phases of the cardiac cycle. However, one, two, or three FPs may still be present in the NT waveforms—in case of a partial influence of the heart dysfunction on the SCG shape—and they might be all the same useful for estimating a subset of CTIs. In our NT group, MC, AO, AC, and MO were separately identified in 9, 12, 8, and 8 waveforms, respectively, corresponding to 26, 35, 25, and 25% of the NT cases.

### Limitations and Perspectives

- In this study, the SCG waveform classification was based on visual inspection. We recognize the subjectivity of the approach but this is the current standard in this field, as we commented at large in the section “Materials and Methods.”

- We estimated the T prevalence in a sample of MI, HF, and TX patients. Further investigations on a wider population, including patients with additional cardiac diseases, would be useful to obtain a more complete picture of the applicability of this methodology in cardiac practice.

- Our results are based on the use of SCG, i.e., the assessment of the *linear* components of precordial vibrations by an accelerometer. Recent findings indicate that information on the *rotational* components of precordial vibrations as obtained by a gyroscope may integrate SCG data and facilitate the identification of CTIs (Jafari Tadi et al., 2017; Sieciński et al., 2020; Shokouhmand et al., 2021). Quantification of the possible benefits of this joint analysis for the CTIs estimation in the clinical practice remains to be explored.

## Conclusion

Our findings clearly indicate that not every cardiac patient has an SCG waveform suitable for the CTI estimation, and even in patients with apparently normal SCG shape, the correspondence between FPs and real valve movements might be inaccurate. Thus, before starting an SCG-based CTI monitoring in a cardiac patient, a preliminary check by a simultaneous SCG-US measure is advisable to verify the applicability of the methodology in the specific subject.

However, it should be highlighted that in patients resulting not suitable for the CTI estimation, SCG analysis can still be useful. Indeed, other indexes of the heart performance, not based on the FP patterns, might be derived from the SCG signal. In literature, different alternative parameters are proposed, based on the estimation of overall signal energy (either obtained from the SCG alone or in combination with data from a gyroscope or the ballistocardiogram) (Morra et al., 2021), and on the evaluation of the overall waveform characteristics through the spectral and time-frequency analysis (Becker et al., 2014; Taebi and Mansy, 2017), dynamic-time feature matching (Zia et al., 2020), hidden Markov model (Wahlstrom et al., 2017), evolving fuzzy neural network (Malcangi et al., 2020), and graph mining algorithms (Inan et al., 2018). All these techniques extract clinical information specifically from the alterations in the waveforms caused by cardiac dysfunction. This means that for the FP identification, the SCG shape distortion is “noise” that may preclude the analysis, while for these techniques, distortion is the main source of information to be exploited.

## Data Availability Statement

The original contributions presented in the study are included in the article/**Supplementary Material**, further inquiries can be directed to the corresponding author.

## Ethics Statement

The studies involving human participants were reviewed and approved by the Comitato Etico della Sezione “IRCCS Fondazione Don Carlo Gnocchi” del Comitato Etico IRCCS Regione Lombardia. The patients/participants provided their written informed consent to participate in this study.

## Author Contributions

ZIZ and MD designed the study, analyzed the data, and prepared the manuscript. All authors contributed to the data collection, reviewed the article, and approved the submitted version.

## Conflict of Interest

The authors declare that the research was conducted in the absence of any commercial or financial relationships that could be construed as a potential conflict of interest.

## Publisher’s Note

All claims expressed in this article are solely those of the authors and do not necessarily represent those of their affiliated organizations, or those of the publisher, the editors and the reviewers. Any product that may be evaluated in this article, or claim that may be made by its manufacturer, is not guaranteed or endorsed by the publisher.

## Footnotes

1
https://www.microdicom.com
